# Non-invasive risk stratification of patients with TTR amyloidosis

**DOI:** 10.1186/1532-429X-15-S1-E112

**Published:** 2013-01-30

**Authors:** Katrin A Scherer, Fabian aus dem Siepen, Rebekka Kammerer, Stefan E Hardt, Ralf Bauer, Uwe Haberkorn, Arnt V Kristen

**Affiliations:** 1 Cardiology, University of Heidelberg, Heidelberg, Germany; 2 Nuclear Medicine, University of Heidelberg, Heidelberg, Germany

## Background

Transthyretin (TTR) is the major constituent of severe systemic amyloid diseases with a broad spectrum of genotypes and heterogeneous phenotypes. Data on risk stratification is lacking. We evaluated the impact of different imaging tools for risk assessment in TTR patients.

## Methods

30 patients (21 male, 9 female; median age 69.5 years) with diagnosis of TTR amyloidosis (wild-type n=13, hereditary n=17) were evaluated by cardiac MRI (CMR; Achieva Intera^®^ Philips Medical Systems, Best, The Netherlands), and ^99m^Technetium-DPD scintigraphy. EDV, ESV, EF and myocardial mass were analyzed on a standard workstation (Philips Viewforum). Longitudinal function was assessed by mitral (MAPSE) and tricuspid (TAPSE) annular plane systolic excursion. Atrial septum thickness was measured on SSFP-4 chamber views. Gadolinium contrast-enhanced CMR (CE-CMR) was assessed semi-quantitatively (absent=0, weak=1, moderate=2, severe=3) in an AHA modified 16 segment model of the left ventricle as well as for the right/left atrium and right ventricle. Nuclear DPD-retention was assessed semi-quantitatively using a region of interest technique by comparison of counts in the heart at 3 hours after injection with whole body counts at 5 min after injection.

## Results

Median troponin T 0.02 [0.004; 0.12] µg/l, NT-proBNP 1217 [13; 6977] ng/l, and MDRD 76 [43; 134] ml/(min*1,73m^2^). By MRI LV mass was 166 [81;354] g, LV ejection fraction 59 [32; 72] %, MAPSE 8 [2; 19] mm, and TAPSE 14 [5; 23] mm). Thickness of atrial septum was 7 [3; 10] mm. Late gadolinium enhancement was present in 26 patients (87%). Mean sum of semi-quantitative CE-CMR was 27.0±2.7 (maximal sum 48). By ^99m^Technetium-DPD scintigraphy heart retention was 6.5 [2.2; 9.5] %, heart-to-body retention was 8.0 [3.5; 12.4] %.

LV ejection fraction and NT-proBNP correlated well with heart and heart-to-body retention. Furthermore, there was a significant correlation of median of LGE and MDRD with heart retention, but with not with heart-to-body retention. LV mass, MAPSE, TAPSE, thickness of interatrial septum as well as modified body mass index did not correlate with heart and heart-to-body retention.

In total, 6 patients died during median survival of 45 (13.6; 79.6) months. Multivariate analysis revealed median of MDRD, LV ejection fraction, and NT-proBNP as independent predictors of survival, but not CE-CMR or scintigraphic heart retention.

## Conclusions

This is the first report comparing different diagnostic tools for risk stratification of patients with TTR amyloidosis. This preliminary study demonstrates the potential impact of cardiac MRI and laboratory findings, but not ^99m^Technetium-DPD scintigraphy for risk stratification of patients with TTR amyloidosis.

## Funding

None.

